# A Fracture in the Proximal Pastern Bone of a Free‐Roaming Giraffe (*Giraffa camelopardalis giraffa*) Under Field Conditions

**DOI:** 10.1002/zoo.70004

**Published:** 2025-07-31

**Authors:** Andri Grobbelaar, Willem Daffue, Collin Albertyn, Francois Deacon

**Affiliations:** ^1^ Department of Animal Sciences, Faculty of Natural and Agricultural Sciences University of the Free State Bloemfontein South Africa; ^2^ Kroonstad Animal Hospital Kroonstad South Africa; ^3^ Absolute Genetics Bloemfontein South Africa

**Keywords:** foot, hoof trimming, injury, pathology, radiograph

## Abstract

Foot and hoof problems are one of the most common health concerns for giraffes (*Giraffa camelopardalis*) held in captivity. However, very limited information is available on the foot pathology for free‐roaming giraffes. A free‐roaming giraffe (*Giraffa camelopardalis giraffa*) situated within a small Free State game reserve presented with limping, and provided a unique opportunity to examine and treat the apparent foot injury. After being sedated and restrained, radiographs were taken in the field. A lateral fracture was diagnosed in the proximal pastern bone of the right front leg of the giraffe. Hoof trimming was used in an attempt to provide treatment and relief to the animal's gait and appearance. The occurrence and treatment of this hoof injury could provide insight on similar pathology in zoo environments.

## Summary

- The first report of a fracture in the pastern bone of a free‐roaming giraffe, including successful intervention and treatment and providing insight on giraffe husbandry.

## Graphical abstract

The field diagnosis and hoof trimming treatment of a pastern bone fracture in a free‐roaming giraffe (*Giraffa camelopardalis giraffa*), offering insight into foot pathology management in wild and captive settings.

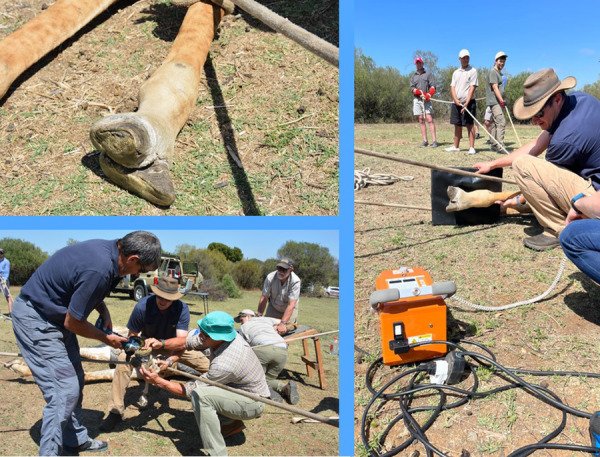

## 1 Introduction

Foot and hoof problems are one of the most common health concerns for giraffes (*Giraffa camelopardalis*) in zoos and have resulted in important and routine husbandry practices for this species held in captivity (Banks et al. 2023; Barta et al. 2006; Burgess 2004; Dadone 2016a, 2016b, 2019; Dadone et al. 2013; Dadone, Schilz, et al. 2016; Gage 2019; Jolly 2003; Lee 1991; Meuffels et al. 2019; Van Zijll Langhout et al. 2018; Wakeman et al. 2014). In contrast to this, little is known about the foot pathology of free‐roaming giraffes, and a low prevalence has been recorded (Dadone et al. 2021). More insight on the pathology and treatment of foot and hoof problems experienced in free‐roaming giraffe could provide valuable insight on the husbandry and management of giraffes held in zoos (Banks et al. 2023; Barta et al. 2006; Burgess 2004; Gage 2019; Jolly 2003; Meuffels et al. 2019).

## 2 Study Area

The presentation of a 2‐year‐old female giraffe (*Giraffa camelopardalis giraffa*) limping in a predator‐free game reserve situated in the Free State, South Africa, provided a unique opportunity to examine and treat the apparent foot injury. The 250 ha game reserve was located on a dolerite hill within the city of Bloemfontein (29°05’56”S 26°14’07”E; 1473 m). The reserve falls within the Bloemfontein Dry Grassland (Gh 5) (Mucina 2014) with a plant species composition consisting of numerous *Olea europaea* and *Buddleja saligna*, with some *Vachellia karroo* and a deteriorated grassland with numerous forb and herbaceous species. Supplementary feeding (e.g., game pellets) was provided on a daily basis to the giraffe population, consisting of four individuals. Other large game species, occurring on the reserve, included an estimated 20 blue wildebeest (*Connochaetes taurinus*), 18 blesbok (*Damaliscus pygargus phillipsi*), 20 European fallow deer (*Dama dama*), 7 nyalas (*Tragelaphus angasii*) and 5 ostriches (*Struthio camelus*).

## 3 Materials and Methods

**Figure 1:**
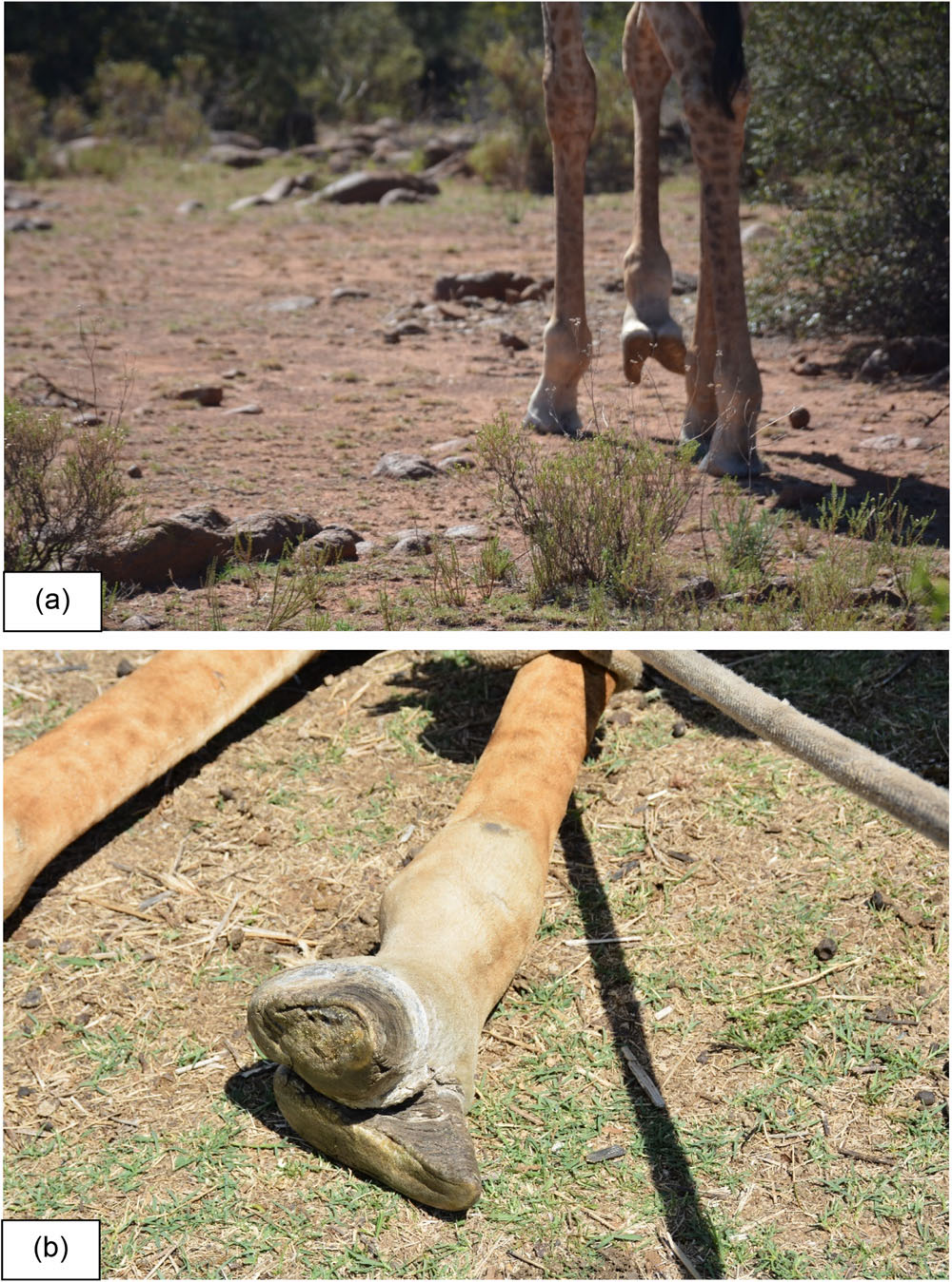
(a) A severe split in the cloven hoof of the right front leg of a 2‐year‐old, free‐roaming giraffe (*Giraffa camelopardalis giraffa*) female at a game reserve in the Free State, South Africa, October 2023, and (b) viewed whilst being restrained during a hoof trimming intervention in the field.

The first observation of the young female giraffe walking impaired was made on 28 October 2023 (Figure 1). Weekly field visits and photographs confirmed the condition, and a decision was made to investigate and treat. The game reserve does not host any natural predators and is frequently visited by members of the public, which prompted swift veterinary intervention.

**Figure 2:**
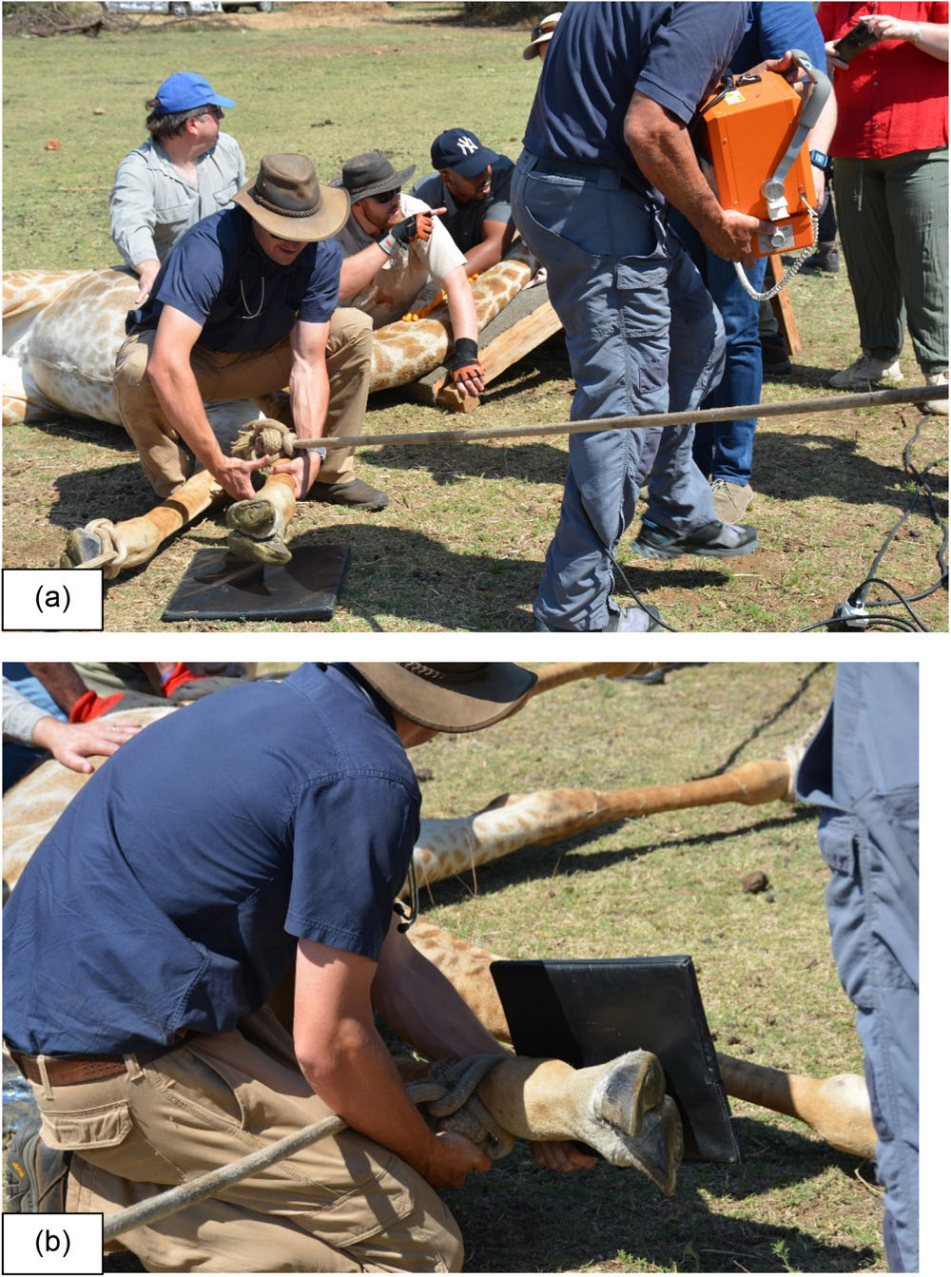
(a and b) Radiographs taken, under field conditions, of the injured right forelimb of a restrained female giraffe (*Giraffa camelopardalis giraffa*) at a game reserve in the Free State, South Africa, in November 2023. Due to the remote location, portable radiographic equipment powered by electric generators were required to facilitate imaging.

Based on earlier work and experience (Deacon et al. 2022), the animal, being an estimated 600 kg, was sedated on 15 November 2023 with 8 mL Thianil (Wildlife Pharmaceuticals Pty Ltd, South Africa) in a single dart. The animal was darted on foot, intramuscularly in the thigh muscle, via a Dan‐Inject dart gun. Once in lateral recumbency, the giraffe was restrained by the capture team, blindfolded and immediately given reversal (2 mL Naltrexone, Wildlife Pharmaceuticals Pty Ltd, South Africa) intravenously. Whilst being manually restrained by means of leg ropes, the front right hoof was inspected visually and a severe split in the cloven hoof was confirmed. Radiographs (Dadone, Olea‐Popelka, et al. 2019; Dadone et al. 2021) were taken and evaluated (Figure 2). A vaginal ultrasound (Lueders et al. 2009) was performed by veterinarians registered at the South African Veterinary Council. The study was approved by the Ethics Committee of the University of the Free State (UFS) and Nature Conservation (reference numbers: AREC12/2011 and AED2015/0066) and formed part of larger research objectives to provide guidelines on successful giraffe immobilisation and capture operations (Deacon et al. 2022).

## 4 Results and Discussion

**Figure 3:**
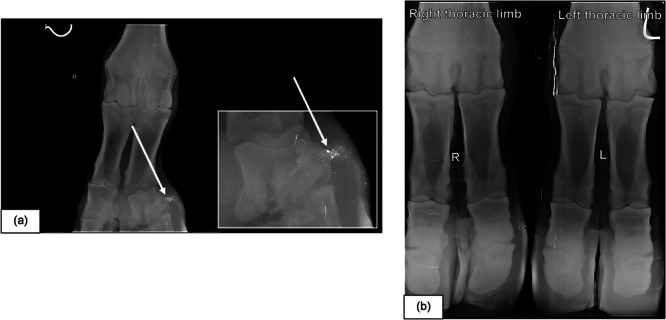
(a) Radiograph illustrating a fracture in the short pastern bone of the right front leg of a 2‐year‐old, free‐roaming giraffe (*Giraffa camelopardalis giraffa*) female at a game reserve in the Free State, South Africa, November 2023 and (b) a radiograph taken of normal, healthy front hooves of a 9‐year‐old, free‐roaming giraffe male at a privately‐owned game reserve in the Free State, South Africa, March 2018.

Radiographs confirmed the occurrence of a lateral fracture in the proximal pastern bone of the right front leg of the giraffe (Figure 3a). When comparing a normal, healthy hoof of another free‐roaming giraffe (Figure 3b), the small lateral fracture in the upper part of the proximal pastern bone becomes evident. Stumbling over a rocky surface or steep slope might be the potential cause of this injury. The fracture had already started to regrow, and it is anticipated that with the passing of enough time, the injury would heal completely.

**Figure 4:**
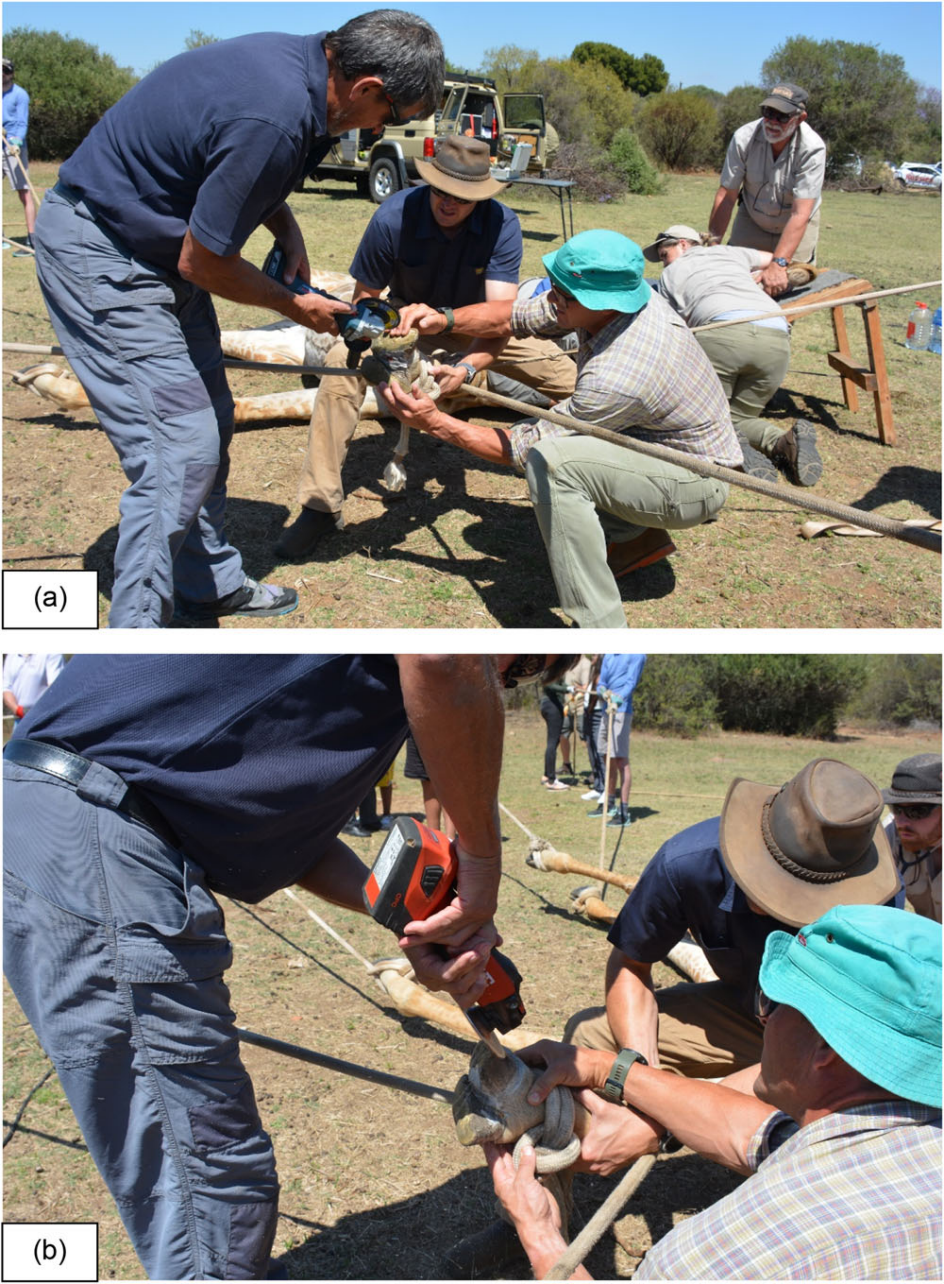
(a and b) Hoof trimming used in an attempt to realign the abnormal hoof growth of the right front leg of a restrained 2‐year‐old, free‐roaming giraffe (*Giraffa camelopardalis giraffa*) female at a game reserve in the Free State, South Africa, November 2023.

**Figure 5:**
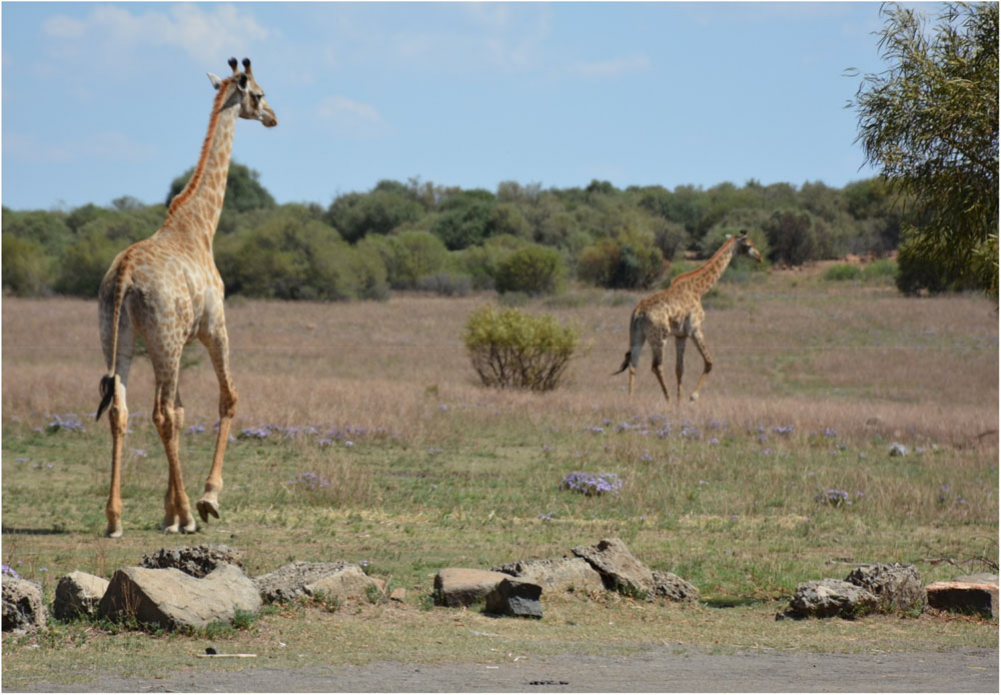
Opportunistic monitoring of the treated 2‐year‐old, free‐roaming giraffe (*Giraffa camelopardalis giraffa*) female (left) showed a gradual improvement in gait and weight‐bearing over subsequent weeks.

Hoof trimming was used in an attempt to realign the abnormal hoof growth and direction to aid in the animal's gait and appearance (Dadone et al. 2018; Van Zijll Langhout et al. 2018; Wakeman et al. 2014) (Figure 4). Precise measurements could not be obtained under field conditions; therefore, the trimming approach was guided by visual assessment of gross asymmetry, apparent weight distribution and overall limb conformation by two experienced wildlife veterinarians. Trimming was performed conservatively and limited to visibly overgrown or imbalanced hoof material, with the primary objective of alleviating abnormal loading forces on the affected limb. Given the free‐ranging status of the animal, repeat interventions were not feasible, and only a single trimming session was conducted. Internal sonar confirmed that the animal was not pregnant at the time, thereby minimising the increase in weight a foetus would have in the following 15‐month gestation period (Wilsher et al. 2013). Following trimming, the animal was monitored opportunistically from a distance by wildlife researchers and showed a gradual improvement in gait and weight‐bearing over subsequent weeks, which is interpreted as a positive clinical response (Figure 5).

## 5 Management Implications

As far as we know, this is the first report of a fracture in the pastern bone of a free‐roaming giraffe, including successful intervention and treatment. As little is known about the foot pathology of free‐roaming giraffes, this intervention and recording thereof contribute to the conservation management of wild giraffes, as well as giraffes held in enclosed environments, such as zoos.

## Conflicts of Interest

The authors declare no conflicts of interest.

## Data Availability

The data that support the findings of this study are available from the corresponding author upon reasonable request.
